# Parenchymal Insults in Abuse—A Potential Key to Diagnosis

**DOI:** 10.3390/diagnostics12040955

**Published:** 2022-04-12

**Authors:** Marguerite M. Caré

**Affiliations:** 1 Department of Radiology and Medical Imaging, Cincinnati Children’s Hospital Medical Center, Cincinnati, OH 45229, USA; marguerite.care@cchmc.org; 2 Department of Radiology and Medical Imaging, University of Cincinnati, Cincinnati, OH 45267, USA

**Keywords:** abusive head trauma, child abuse, hypoxic-ischemic injury, computed tomography, magnetic resonance imaging

## Abstract

Subdural hemorrhage is a key imaging finding in cases of abusive head trauma and one that many radiologists and radiology trainees become familiar with during their years of training. Although it may prove to be a marker of trauma in a young child or infant that presents without a history of injury, the parenchymal insults in these young patients more often lead to the debilitating and sometimes devastating outcomes observed in this young population. It is important to recognize these patterns of parenchymal injuries and how they may differ from the imaging findings in other cases of traumatic injury in young children. In addition, these parenchymal insults may serve as another significant, distinguishing feature when making the medical diagnosis of abusive head injury while still considering alternative diagnoses, including accidental injury. Therefore, as radiologists, we must strive to look beyond the potential cranial injury or subdural hemorrhage for the sometimes more subtle but significant parenchymal insults in abuse.

## 1. Introduction

Child physical abuse and neglect fatality rates have continued to increase over the past several years, with almost 70% of fatalities involving children less than age 3 years and just under half occurring in infants and young children less than age 1 year [1]. Abusive head injury is a serious form of child physical abuse and is the leading cause of deaths in physically abused children [2]. In survivors, it often results in significant morbidity with permanent neurologic and developmental disabilities [3,4]. Even those patients with apparent minor injuries at presentation may be left with long-term altered behavioral or academic outcomes [4].

Neuroimaging plays a key role in the diagnosis and subsequent evaluation of children with abusive head injury. Since patients may present with little to no outwards signs of trauma, it is imperative that radiologists be familiar with the imaging findings, reported controversies [5], as well as correlate the imaging with the clinical and historical presentation while also contemplating alternative explanations, such as infections, metabolic and bleeding disorders, and especially accidental injury. Although child abuse pediatricians are usually familiar with the imaging features of abusive head injury and child physical abuse, in my experience, I find that radiology trainees often have limited knowledge of the imaging findings beyond subdural hemorrhage and some of the more classic skeletal injuries. Although sometimes simplified and referred to as the “triad” of findings in abusive head injury, which includes subdural hemorrhages, retinal hemorrhages, and encephalopathy, these patients can be complex, with the medical diagnosis of abusive head trauma [5,6] not only limited to this “triad”. However, these features remain key for arriving at a potential diagnosis [7].

Subdural hemorrhage is the most frequent intracranial finding in abusive head injury [8,9,10,11] and is reported to occur in over 80% of cases [10,11,12,13]. Post-traumatic subdural hemorrhage occurs more commonly following abusive rather than accidental head injuries in infants and young children [14,15,16,17,18,19,20,21,22]. In a systematic review identifying features that aid in distinguishing abusive from accidental head injuries in young children, Kemp et al. [22] found subdural hemorrhage to be significantly associated with abusive head injury, potentially serving as a marker of trauma when a child presents with unexplained head injuries or injuries that are out of proportion to only minor trauma reported by a caretaker. In addition to subdural hemorrhage, multifocal or diffuse brain parenchymal insults in patterns suggesting hypoxic-ischemic brain injury and cerebral edema were also identified as key features favoring abusive rather than accidental head injury [22]. In abused patients, these diffuse parenchymal insults are accompanied by subdural hemorrhage but are less commonly seen in children presenting with fractures [23]. Clinically, these patients more commonly present with an increased frequency of seizures, requirement of intubation [24], and hypotension/hypoperfusion and/or hypoxia [23] in comparison to abused patients with more focal brain injury.

These diffuse parenchymal insults may be elusive and difficult to detect on early CT imaging, the modality most frequently utilized for initial evaluation. Therefore, it is imperative that radiologists look beyond the potential cranial injury and extra-axial hemorrhage that might serve as a marker for trauma in these young patients, searching for these parenchymal insults that may be very subtle and potentially missed on initial imaging but are frequently the most devastating and may serve as a means to help distinguish inflicted from accidental head injury [22]. In addition, they strongly contribute to the significant morbidity and numerous fatalities observed in abusively head-injured patients [22,24,25,26,27]. Because of these reasons, in my practice, I stress to the radiology trainees the importance of recognizing these parenchymal insults and encourage them to take a second look beyond the often more obvious subdural hemorrhage.

## 2. Patterns of Diffuse Parenchymal Insults

Diffusion-weighted imaging (DWI) is a key sequence in magnetic resonance imaging (MRI), and its indications have reached far beyond its early application in detecting cytotoxic edema in the clinical setting of stroke. In cases of abusive head injury, DWI is invaluable in the detection of associated brain parenchymal injuries and has been shown to demonstrate more extensive parenchymal injury [26,28] than might be detected on conventional T1, T2, or FLAIR sequences. Several authors [24,25,26,27,28,29] have evaluated the utility of DWI in abused infants and described various imaging patterns that may be observed, including diffuse hemispheric involvement of the brain, the most commonly observed pattern. Although presumed to be at least partially hypoxic-ischemic in etiology, the precise nature and pathophysiology behind this pattern of diffusion restriction in this population of infants and young children remains incompletely understood and debated. A recent letter to the editor by Silverman et al. [30] challenged the use of the term hypoxic-ischemic injury (HII) when describing the often-large areas of parenchymal abnormality and associated diffusion restriction frequently seen on MR imaging in abusive head injury. The authors advocate for a more basic terminology, cytotoxic edema, as this avoids the assertion that the underlying cause of parenchymal injury in these patients is just hypoxic-ischemic injury. However, Orru’ et al., as pointed out in their response, described multiple injury patterns in their initial study [27], including those that result from direct injury to the brain (contusions and axonal injury) that also result in cytotoxic edema on MRI. Hence, at this point in our understanding, although the exact mechanism of injury at a cellular level might be incompletely understood as well as why some abused children present with this diffuse, hemispheric pattern of injury [23] while others do not, the injury, at least in part, is thought to be hypoxic-ischemic [24,25,26,27,28,29,31,32,33]. Argument aside, this discussion and lack of complete understanding of this complex process, however, should not detract from the necessity of physicians to recognize these parenchymal insults as being present in abusive head trauma patients.

**Figure 1:**
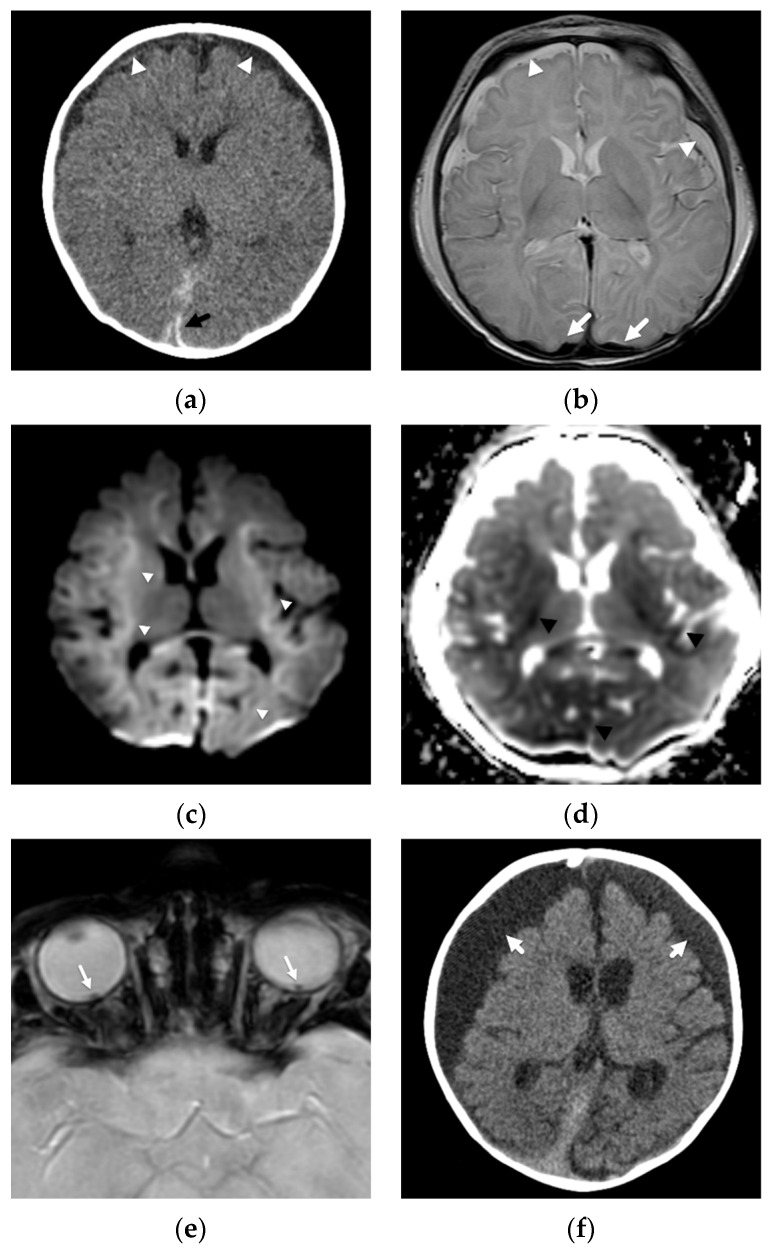
Subdural hemorrhage and subdural collections in an unresponsive 2-month-old male presenting with forehead bruising and respiratory depression. (**a**) Axial noncontrast head CT image demonstrates thin, high attenuation subdural hemorrhage along the posterior left occipital lobe (black arrow) and bilateral, low attenuation frontal subdural collections (white arrowheads). There is also subtle loss of gray-white matter differentiation bilaterally; (**b**) axial T2-weighted MR image on day 4 shows regions of decreased gray-white matter differentiation throughout both cerebral hemispheres with T2 hyperintense subdural collections (white arrowheads) and hypointense subdural hemorrhage posteriorly (white arrows); (**c**) axial b-1000 diffusion-weighted image demonstrates diffuse areas of abnormal, restricted diffusion (white arrowheads) in both cerebral hemispheres consistent with cytotoxic edema/hypoxic-ischemic injury; (**d**) apparent diffusion coefficient image shows corresponding regions of diffusion restriction predominantly in cortical and subcortical regions of both cerebral hemispheres (black arrowheads); (**e**) axial susceptibility-weighted image shows small, bilateral retinal hemorrhages (white arrows), confirmed clinically; (**f**) axial CT image at 2 month follow-up show diffuse brain parenchymal volume loss with now large, bilateral subdural collections (white arrows).

These diffuse, hemispheric patterns of injury/insult are observed in abusive head injury much more commonly than in cases of accidental pediatric head trauma [22,24], suggest permanent brain injury, and portend a poor outcome [22,23,24] (Figure 1). Except in cases of significant accidental trauma, such as pedestrians hit by cars or motor vehicle accidents, this type of injury is not found in children with more routine, household injuries [34], such as a short fall from a couch. In a comparative study looking at DWI in young children with abusive (*n* = 30) and accidental (*n* = 22) injuries, Ichord et al. [24] found that patients in their series with abusive injuries much more commonly demonstrated a pattern of restricted diffusion concerning for hypoxic-ischemic injury than those suffering accidental injuries (37% versus 9%). Nine of thirty abused patients demonstrated a predominantly diffuse pattern of parenchymal injury with a coexistent, small-volume subdural hemorrhage or other traumatic lesion compared with only 1/22 of accidental cases. Their patients with these diffuse insults tended to be younger in age, had greater acute and chronic neurologic abnormalities, and a higher likelihood of requiring longer-term inpatient rehabilitation.

**Figure 2:**
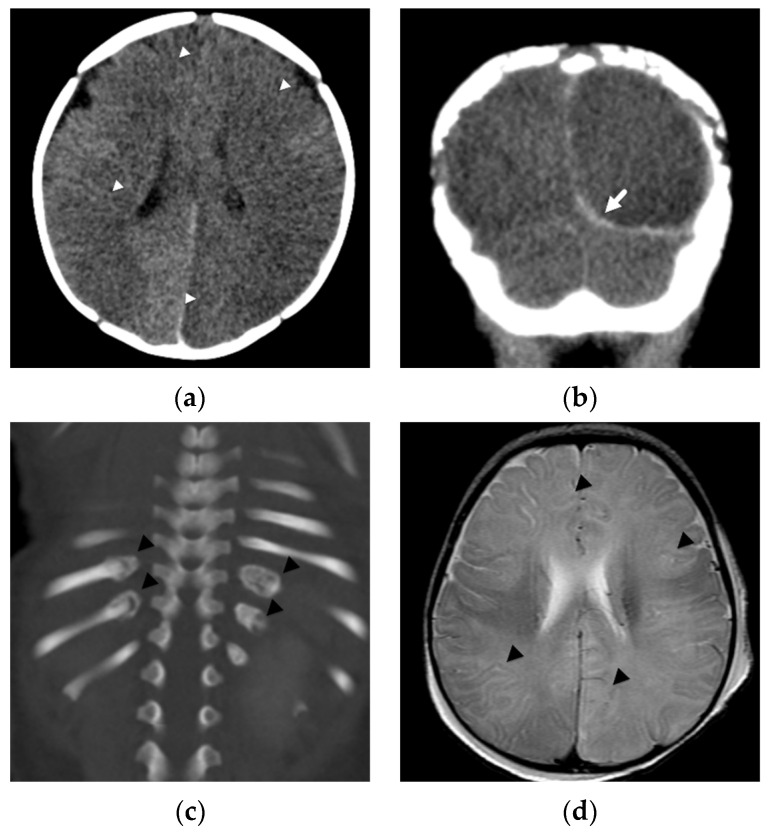

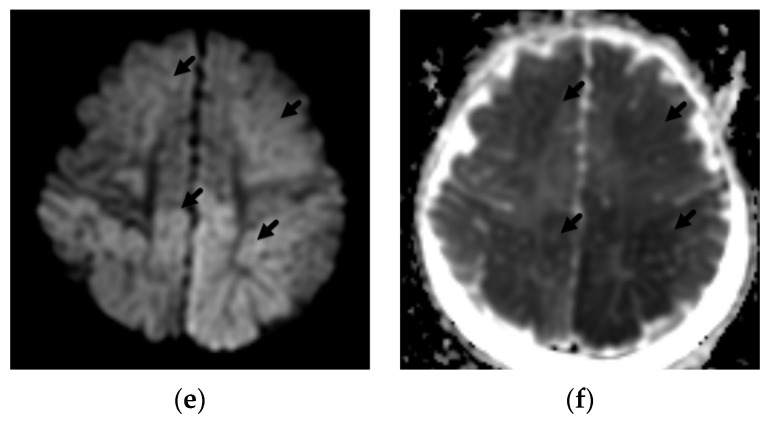
Diffuse, bilateral hemispheric attenuation abnormality in a 3-month-old male presenting in status epilepticus with multifocal facial, extremity, and trunk bruises. (**a**) Initial axial noncontrast head CT image with diffuse, abnormal attenuation throughout both cerebral hemispheres (white arrowheads); (**b**) coronal noncontrast CT image shows thin subdural hemorrhage along the left tentorial leaflet (white arrow); (**c**) coronal reconstruction bone algorithm image from an abdominal CT at presentation shows healing, bilateral posterior rib fractures (black arrowheads); (**d**) axial T2-weighted image on day 4 demonstrates diffuse, hemispheric loss of gray-white matter differentiation (black arrowheads); (**e**) axial b-1000 diffusion-weighted image demonstrates diffuse areas of abnormal, restricted diffusion (black arrows) in both cerebral hemispheres consistent with cytotoxic edema/hypoxic-ischemic injury; (**f**) apparent diffusion coefficient image shows corresponding regions of diffusion restriction predominantly in cortical and subcortical regions of both cerebral hemispheres (black arrows).

In another study, Zimmerman et al. [25] evaluated DWI in 33 abused infants and young children. The most common pattern of parenchymal insult, found in 13/33 (39%) patients, was of diffuse, restricted diffusion of the supratentorial cortex and white matter. Combined with their second most common pattern, which was present in 12/33 (36%), that of a more watershed or border zone distribution of restricted diffusion with primarily supratentorial but also infratentorial involvement, almost three-quarters of their patients had parenchymal imaging patterns suggesting diffuse hypoxic-ischemic injury/insult and resulting in permanent brain injury. These areas of restricted diffusion were not limited to isolated vascular distributions and were more extensive than focal impact or contusional injuries Figure 2.

Kadom et al. [29] evaluated 64 young children initially referred for suspected abuse with both cervical and brain MRI studies. Forty-five percent (29/64) of the total cohort had brain parenchymal diffusion abnormalities on MR. Fifty-five percent with diffusion abnormalities on MR (16/29) had a pattern suggesting a diffuse and symmetric hypoxic-ischemic insult, with 12/16 (75%) of those having a coexistent cervical spine injury. Abusive injury was diagnosed in 88% (14/16) of cases with a bilateral and diffuse parenchymal pattern and in 10/12 (83%) of cases with this parenchymal pattern and coexistent cervical spine injury. This coexistence of spinal injuries on MR, including spinal subdural hemorrhage, has been demonstrated by multiple other authors [35,36,37,38] and is likely being increasingly recognized on imaging, as recent trends advocate for whole-spine MRI in children presenting with concerns of abusive head injury.

In a more recent investigation, Orru’ et al. [27] reported on 57 abused patients less than age 5 years and described two predominant patterns of parenchymal injury including large areas of primarily cortical and subcortical restricted diffusion or a diffuse cortical and deep gray matter insult, each consistent with cytotoxic edema and presumed hypoxic-ischemic injury and present in 70% of their DWI-positive patients (36.8% of the total abuse cohort). In-hospital deaths occurred in twenty percent of cases (6/30) with diffusion abnormalities, all with diffusion restriction/cytotoxic edema patterns suggesting hypoxic-ischemic injury. In contrast, however, there were no fatalities in patients with more focal diffusion abnormalities or in cases lacking parenchymal injury. In contrast to other studies [24,25,29], an asymmetrical pattern of parenchymal insult involving bilateral cortical and subcortical white matter occurred more commonly than a diffuse but more symmetrical hemispheric pattern of restricted diffusion. None correlated with a single-vessel vascular distribution infarct.

McKinney et al. [39], in reviewing 53 children (11/53 highly suspicious for AHT) less than age 3 years with acute traumatic head injury, described an asymmetrical pattern of presumed hypoxic-ischemic injury (HII) with small-volume subdural hemorrhage. Four of fifty-three cases overall had HII on CT or MR, with 3/4 of cases (75%) highly suspicious for abusive head trauma. Of these, two had a primarily unilateral pattern of hemispheric insult with small-volume subdural hemorrhage and no associated skull fracture. Although the etiology of this unilateral pattern of insult is incompletely understood, in both of their cases, no arterial abnormalities were found on dedicated MR imaging with magnetic resonance angiography (MRA), and the distribution in each case extended beyond typical, isolated anterior or posterior distribution infarcts.

**Figure 3:**
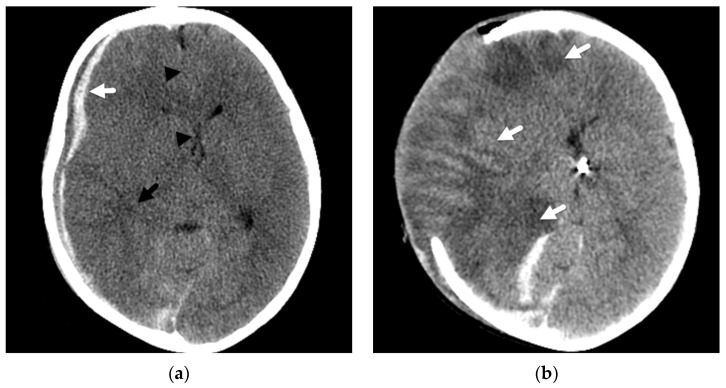
Mixed attenuation right subdural hemorrhage in a 3-year-old female fatality presenting with abrupt mental status change and multifocal bruises after a reported fall. (**a**) Initial axial noncontrast head CT demonstrates a mixed attenuation subdural hemorrhage (white arrow) with mass effect, midline shift (black arrowhead), and effaced right temporal horn (black arrow); (**b**) next-day CT image shows changes of a decompressive hemicraniectomy with diffuse, abnormal attenuation of the right cerebral hemisphere (white arrow), which herniates through the cranial defect.

In another abusive head injury study, Foster et al. [23] described that when this unilateral pattern of hemispheric involvement was observed, coexistent subdural hemorrhage was located either bilateral or ipsilateral to the parenchymal insult but not solely contralateral in distribution [23], and the associated mass effect was often out of proportion to the volume of subdural hemorrhage (Figure 3). Some authors [40,41] have suggested that this constellation of predominantly unilateral parenchymal insult with associated SDH has similarities to a rare entity described in young athletes suffering repetitive trauma/concussions and might result from cerebral dysautoregulation. However, this remains uncertain and controversial [42], especially in reference to this younger pediatric population. As with cases presenting with a large volume subdural hemorrhage resulting in significant mass effect, these patients may undergo a decompressive hemicraniectomy in an attempt to relieve mass effect and midline shift, thus potentially alleviating subsequent infarction to areas of the contralateral brain that are more typical of herniation effects.

The exact etiology of this extensive, bilateral or unilateral restricted diffusion on MR or corresponding findings on CT remains incompletely understood although is likely multifactorial with potential contributions from apnea [43], hypoperfusion and loss of vascular autoregulation, neuroinflammation [44], and excitotoxic mechanisms resulting in secondary neuronal injury [45,46]. This incomplete understanding may be, in part, a result of the inability to routinely have detailed histological studies performed on each child fatality [47] as well as the varying traumatic mechanisms of injury and timing of a death in relation to the initial insult. A two-part publication in 2001 by Geddes et al. [31,32] described the histologic evaluation of 53 AHT fatalities. Diffuse hypoxic-ischemic neuronal injury was found most frequently on evaluation, being present in 77% of total cases and in 84% of infants, thus emphasizing its potential role in parenchymal injuries in abuse. In contrast, despite the presumed traumatic nature of the fatalities, widespread traumatic axonal injury was found infrequently, present in only 3/53 cases, two of which were infants having suffered severe cranial injuries. However, cervical epidural hemorrhage and more localized axonal injury near the lower brainstem and craniocervical junction were found in 11 study infants, but these were not present in age-matched controls, potentially indicating a role for respiratory depression or compromise as a cause of the more diffuse hypoxic-ischemic neuronal injury, as apnea was listed as a clinical feature in 75% of their abuse cases.

More recently, Matschke et al. [33] reviewed histopathology and/or autopsy data of 50 abusive head trauma cases, almost half being fatal within the first 24 h. None clearly had diffuse, traumatic axonal injury. However, in select cases with immediate death, localized traumatic axonal injury in the brainstem was observed close to known respiratory centers. All cases had findings indicating hypoxic-ischemic injury.

## 3. Imaging of Diffuse Insults

On initial CT imaging, these diffuse or asymmetric patterns of parenchymal injury may be difficult to perceive but have been reported to be present as early as 72 min from the insult [11]. Findings may include only subtle loss of the gray-white matter junction and parenchymal hypoattenuation, progressing to more visible, abnormal attenuation over a period of hours to days [11]. I often find it helpful to review the images on a 9 on 1 display, like radiologists reviewed CT cases prior to picture archiving and communication systems (PACS). Like seeing a finding from the back of the room, this image display often aids in the detection of subtle gray-white matter differentiation loss and the ability to detect sulcal effacement. In addition, on early imaging, the ventricles and extra-axial spaces may be preserved, making detection of subtle changes even more difficult. Progressive brain swelling may ensue and may be out of proportion to coexistent subdural hemorrhage. There may be involvement of both cortical and deep gray matter, subcortical and deep white matter, as well as the supratentorial and infratentorial brain. Extent of the injury as well as the timing of the imaging in relation to the insult can alter the conspicuity and distribution of imaging abnormalities, including the distribution of diffusion restriction, as many of these patients may be too unstable to undergo early MR imaging. However, once the child is stabilized, brain MRI with diffusion-weighted imaging will be key in further delineating the extent of parenchymal injury in these patients.

## 4. Focal Parenchymal Insults

Additional parenchymal injuries may occur in abusive head injury as a direct/primary result of the traumatic mechanism although these too may evolve with ongoing secondary injury [48]. These injuries primarily include traumatic or diffuse axonal injury, parenchymal contusions, and lacerations. Unlike the diffuse hemispheric insults, these focal parenchymal insults are not a significant distinguishing feature for abusive head injury [22] although combinations of injuries, including spinal injuries, may coexist. Moreover, patients with more focal parenchymal insults less commonly present with significantly altered mental status [22,23,24].

Traumatic or diffuse axonal injury (DAI) is found frequently following severe accidental trauma, such as bicycle accidents, high-speed motor vehicle collisions, and pedestrians hit by motor vehicles. However, this pattern of injury is infrequently observed on imaging [22,24,25,26,27] or at dedicated histopathologic evaluation [31,32,33] in young pediatric patients suffering inflicted injuries. In MR studies evaluating different patterns of diffusion restriction in abusive head injury, Zimmerman et al. [25] observed DAI in only 2/33 cases. Ichord et al. [24] observed traumatic axonal imaging in only 1/30 cases of inflicted trauma and in 1/22 similarly aged accidental cases, each based on a typical distribution of axonal injury in the frontal subcortical white matter and corpus callosum, respectively. Orru’ et al. [27] characterized 5/30 DWI-positive cases of AHT as traumatic axonal injury, each patient 3 months of age or younger, as they observed small, linear, or punctate foci of diffusion restriction in the corpus callosum or near the gray-white matter junction, again, typical locations for diffuse axonal injury. The infrequent finding of diffuse axonal injury in abused infants and young children has been the experience at my institution as well. Although infrequently observed on imaging studies, traumatic axonal injury near the craniocervical junction and involving the lower brainstem in autopsy subjects [32,33], particularly in younger, abused infants, has been demonstrated and hypothesized to be the potential source of the respiratory depression and apnea frequently observed in this population, thus potentially contributing to associated secondary hypoxic-ischemic injury in the more global brain.

**Figure 4:**
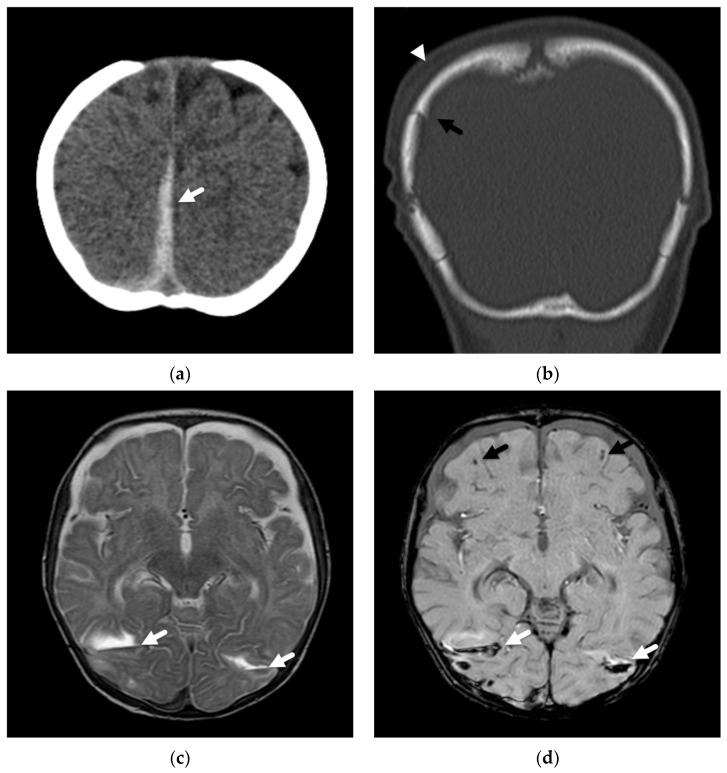
Axonal injury and lacerations/contusional tears in a 4-month-old male presenting with seizure and lethargy. (**a**) Initial axial CT image demonstrates right parafalcine high-attenuation subdural hemorrhage (white arrow); (**b**) coronal reconstruction in bone algorithm demonstrates a right parietal bone fracture (black arrow) with overlying soft tissue swelling (white arrowhead); (**c**) axial T2-weighted MR image on day 4 demonstrates bilateral lacerations/contusional tears with fluid-hemorrhagic levels near the temporal-occipital lobe junctions (white arrows); (**d**) axial susceptibility-weighted image demonstrates layering hemorrhage in lacerations/contusional tears (white arrows) and axonal injury near the cortical-white matter junction in each frontal lobe (black arrows).

Axonal injuries may be very subtle on CT imaging but may appear as multiple, punctate, low-attenuation foci or petechial hemorrhages, usually involving hemispheric white matter, the corpus callosum, cerebellum, and brainstem. Deep subarachnoid hemorrhage and brain swelling may be seen as associated findings on both CT and MRI. Susceptibility-weighted imaging is a key MR sequence for detecting the punctate hemorrhagic foci, and diffusion-weighted imaging aids in the detection of non-hemorrhagic, traumatic axonal injury Figure 4.

**Figure 5:**
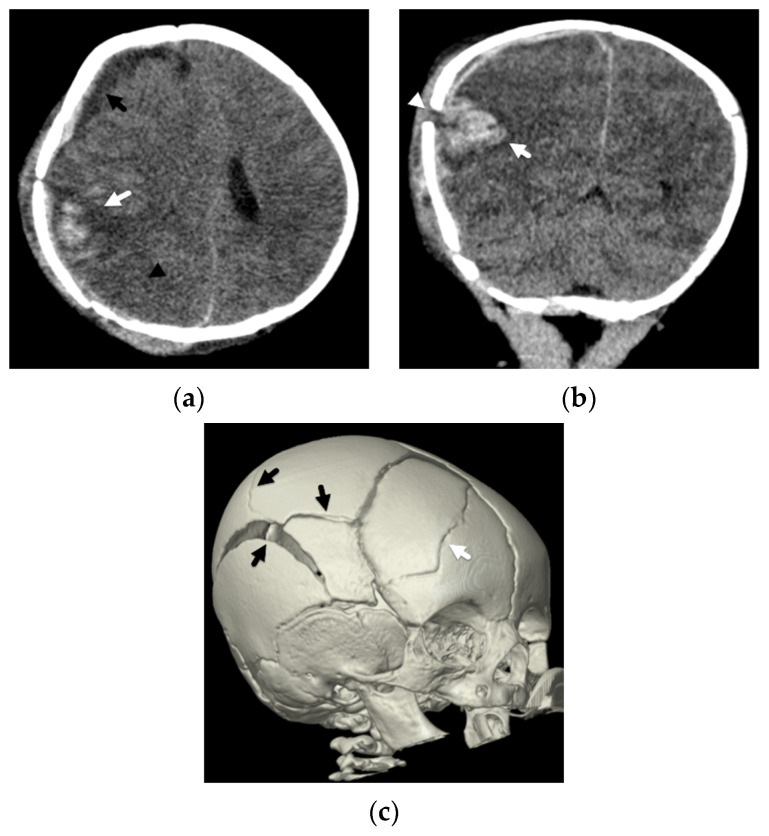
Skull fracture and hemorrhagic contusion in a 2-month-old female presenting with the history of a fall off a couch. (**a**) Initial axial CT demonstrates a hemorrhagic contusion at the posterior temporal-parietal lobe junction (white arrow) with mixed but predominantly low-attenuation hemorrhagic subdural (black arrow). More focal loss of gray-white matter differentiation (black arrowhead) may also suggest coexistent venous infarction or bland contusion; (**b**) coronal CT reconstruction shows the hemorrhagic contusion (white arrow) deep to a diastatic right parietal bone fracture (white arrowhead); (**c**) three-dimensional reconstruction of the skull demonstrates a mildly complex right parietal bones fracture (black arrows) and right frontal bone fracture (white arrow).

Traumatic head injury may also result directly in parenchymal contusions and lacerations. These injuries are also less commonly observed in abusive head trauma [24,25,26,28] but, when present, are frequently seen in conjunction with other traumatic injuries, such as overlying extra-axial hemorrhage or fractures (Figure 5). Given their more focal nature, these insults typically result in more localized neurologic deficits instead of a diffuse encephalopathy, although they certainly can be seen in conjunction with more diffuse injuries. In Zimmerman et al.’s review of DWI in abusive head trauma [25], parenchymal contusions were one of the least frequent patterns of described parenchymal injury, occurring in only 2/33 cases, potentially reflecting different mechanisms of injury in the abusive head injury population as opposed to a direct cranial insult from an accident, such as when a child accidentally falls from a second-story window. Other studies have also demonstrated the more infrequent occurrence of contusions in this population [24,27]. Contusions more commonly affect older children, are often multiple, maybe hemorrhagic or non-hemorrhagic, and are usually centered near the surface of the frontal and temporal lobes near the adjacent bone surfaces and dural reflections. Multiplanar CT reconstructions will allow increased detection of subtle contusions in the inferior frontal and anterior temporal lobes. On MRI, gradient echo or susceptibility-weighted imaging will aid in detection of more subtle hemorrhagic contusions, while non-hemorrhagic contusions may be more readily detected on diffusion-weighted imaging along the surface of the brain. Contusions often increase in size over the first few days as well as develop surrounding edema.

**Figure 6:**
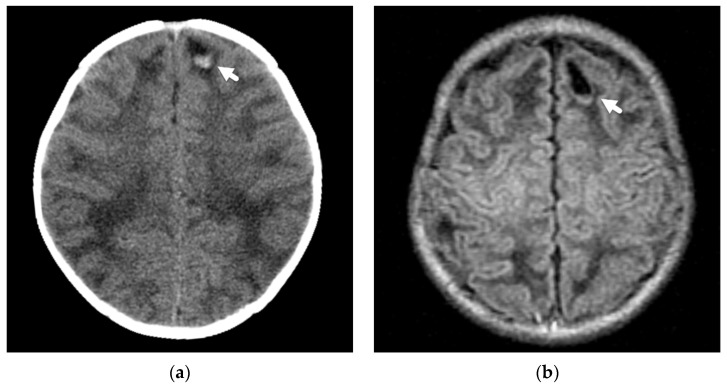

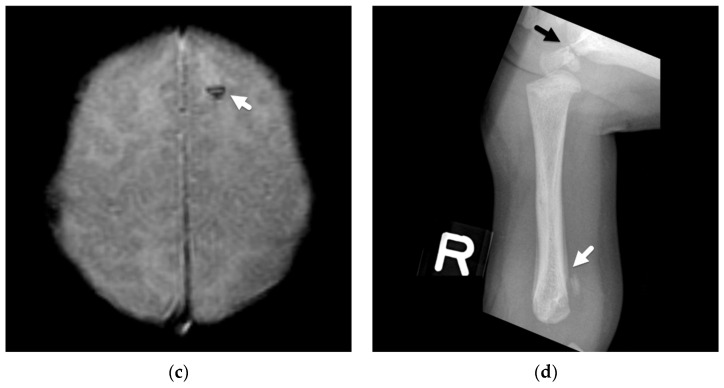
Focal laceration or contusional tear in a 2-month-old former preterm male infant presenting with seizure. (**a**) Initial axial noncontrast CT demonstrates a small, focal, left frontal lobe laceration/tear with layering posterior hemorrhage (white arrow); (**b**) axial T1-weighted image shows a hypointense, well defined laceration/tear with subtle layering hemorrhage posteriorly (white arrow); (**c**) axial multiplanar gradient recalled acquisition in the steady-state (MPGR) image shows the layering posterior hemorrhage (white arrow); (**d**) follow-up skeletal survey demonstrates healing right acromion fracture (black arrow) and distal humeral periosteal reaction (white arrow) suggesting a healing fracture.

Slit-like lacerations or tears/clefts at the cortical-white matter junction, mainly in the frontal and anterior temporal lobes, have been described in young infants as a result of trauma. On imaging, these lesions may appear as focal cerebrospinal fluid-like clefts or lesions with layering fluid–fluid levels or layering hemorrhage [47,49,50] (Figure 6). Palifka et al. [50] described these focal lacerations in 18/137 abusive head injury patients of less than 3 years of age, with almost 90% (16/18) occurring in patients less than 1 year of age. However, none were demonstrated in a comparison cohort of patients with moderate to severe accidental head injury (*n* = 28 with 8 less than 1 year of age), including those that sustained injuries from falls, motor vehicle collisions, and young pedestrians hit by automobiles. In their series, the lacerations or tears were detected on noncontrast CT in 9/18 cases as linear or fluid/hemorrhagic attenuation clefts in the subcortical white matter in the supratentorial brain, with sixty percent (11/18) demonstrating associated fractures or regional scalp swelling. MR imaging demonstrated a linear cleft or focal fluid-fluid level or hemorrhagic level in the subcortical white matter and in over half, was best demonstrated on gradient echo or susceptibility-weighted sequences. In 2/18 patients, however, these lacerations were most easily detected on DWI. Lesions most commonly occurred in the frontal lobes, and although some were isolated, seven patients had multiple lacerations. In most cases, other traumatic lesions, including retinal and extra-axial hemorrhages or injuries to other extracranial locations, were detected either clinically or on imaging.

Similar slit-like tears or clefts in young infants near the cortical-white matter junction, predominantly in the anterior temporal and frontal lobes, have been described in the perinatal period, especially following instrument-assisted deliveries [51,52]. Although these cerebrospinal fluid-like clefts may, in some ways, resemble small areas of cystic encephalomalacia, careful evaluation on imaging should help confirm their characteristic appearance and eventual evolution over time. In the absence of prior documented trauma and when observed on imaging in young infants, these lacerations or clefts should raise concerns for potential inflicted head injury [49,50].

Another less commonly encountered pattern of parenchymal injury in abuse is that of a venous infarction [24,25], found in 4/33 cases in one series. This pattern should be suspected with more focal attenuation abnormality on CT or diffusion restriction on MR deep to an injured/avulsed bridging vein or subdural hemorrhage but also when other traumatic lesions are seen in association with parenchymal hemorrhages [24] and are identified in typical venous vascular distributions, such as the parasagittal frontoparietal locations and the posterior temporal lobes [53]. These imaging findings may overlap or be difficult to distinguish from parenchymal contusions. In general, large parenchymal hemorrhages are infrequently seen in abusive head trauma. However, cases presenting with rapid expansion of hemorrhage may suggest the development of an underlying coagulopathy, as acquired coagulopathies have been reported as a complication of inflicted head injury [54].

## 5. Imaging Evaluation

As with other causes of acute traumatic head injury in pediatric patients, CT remains the initial imaging modality of choice when an infant or young child presents with suspicions of abusive injury [55,56]. Additional guidelines for head imaging based on clinical examination findings, modality appropriateness stratification, and information regarding relative radiation level per modality can be found under the American College of Radiology Appropriateness Criteria [56] as well as supplemented by more local hospital or institutional guidelines. As with imaging all pediatric patients, CT imaging should be performed utilizing pediatric dose-reduction techniques and parameters [57].

Although MRI with DWI is invaluable in evaluating these young brains, it remains paramount that radiologists scrutinize head CT examinations for not only cranial injuries and/or extra-axial/subdural hemorrhage but also for the presence of these potential parenchymal injuries, including cytotoxic edema/hypoxic-ischemic injury. In severe cases with fatalities, the initial CT may be the only head imaging performed prior to death. Multiplanar and 3-dimensional (3D) CT reconstructions of the head can be routinely generated without additional radiation exposure to the child. These aid in evaluation of craniocervical junction hemorrhage [58] and injury, identifying small posterior fossa and convexity subdural hemorrhages [59] and potential bridging vein injury or thrombosis over the cerebral convexities [60,61] as well as providing more optimal evaluation of the pediatric skull [62,63]. In my practice, in addition to the axial standard and bone algorithm images, we routinely reconstruct and send 3D bone reconstructions as well as coronal and sagittal reconstructions in both standard and bone algorithm. CT imaging is rapid, often alleviating the need for sedation, and it can be used to evaluate for potentially coexistent spinal, thoracic, and abdominal injuries.

MRI should be considered and is frequently utilized in the non-acute setting or when the child is stabilized to further evaluate the brain as well as to assess for potential spinal injuries in patients presenting with suspicions of abusive head injury [55,56]. Diffusion-weighted imaging is invaluable for detecting brain parenchymal injury [55], especially the hemispheric insults with cytotoxic edema/hypoxic-ischemic injury, and should routinely be included when evaluating this population of infants and young children. In addition, standard T1- and T2-weighted sequences provide increased anatomic detail over CT. On T2-weighted imaging of the brain, look for subtle loss of the normal cortical ribbon, often well-seen in infants due to the lack of myelinated white matter. Fluid attenuated inversion recovery (FLAIR) sequences may be considered but, at my institution, are not routinely performed in infants due to the unmyelinated white matter. However, as with the first-echo T2 images, FLAIR sequences may aid in detection of small subdural collections or hemorrhage [64]. As with other trauma protocols, multiplanar gradient recalled acquisition (MPGR) or susceptibility-weighted sequences should be included to assess for the presence of blood products [56].

To avoid radiation associated with CT, some authors have advocated for the use of initial MRI instead of CT in patients less than 24 months of age presenting with suspicions of abuse but lacking neurologic symptoms [57]. Although the length of an MRI may preclude evaluation of a child without the use of and potential risks of anesthesia [65,66], imaging using more rapid imaging protocols and sequences have been suggested and may be a feasible alternative in young children presenting with concerns of traumatic head injury [67,68,69]. However, other studies have described limitations associated with these techniques [70,71] in the setting of suspected trauma, including lack of 24 h MRI coverage at many institutions. Currently, at my institution, CT remains the initial imaging modality of choice in this population.

Severity and timing of the head imaging in relation to presentation and insult may alter the conspicuity of findings on both CT and MRI. Therefore, serial imaging should be considered to more thoroughly demonstrate the imaging abnormalities but also to potentially provide improved imaging correlation with the historical and clinical examination findings while keeping in mind potential limitations in timing abusive injuries based on imaging alone [11,72].

## 6. Clinical and Surgical Considerations and Outcomes

Treating physicians and radiologists not only need to be vigilant in their acute management of pediatric head trauma patients, but they also need to remain keenly aware that abusive head trauma patients may present with rather nonspecific clinical features, such as irritability or lethargy, that may not, at least initially, point directly to a traumatic etiology. Historical “red flags” may potentially alert treating physicians and thus cause them to consider abusive head injury in the potential differential. These may include when a child presents with clinical or radiographic signs of head injury but with no or with a changing history of trauma, reports of only minor trauma out of proportion to what is observed or inconsistent developmentally, or when the caretaker blames findings on rescue or resuscitation efforts that occurred prior to hospital arrival [73,74]. A multidisciplinary approach to these cases is necessary to acutely manage the patient but also to provide subsequent evaluation and diagnosis as well as continuing assessment and rehabilitation beyond the inpatient period.

In the acute period, neurosurgical intervention may be necessary to evacuate a large subdural hemorrhage or decompress the brain in cases of significant brain swelling or elevated intracranial pressure in an attempt to alleviate progressive brain herniation or insult and has been shown to be performed more frequently in cases of abusive rather than accidental head trauma [75,76]. In a comparative study [76] evaluating decompressive craniectomies performed in both accidental and abusive head injury cases for mass lesions and elevated intracranial pressure, mortality rates were significantly higher in abusive head trauma cases despite the surgical decompression. Additionally, abused patients were more likely to have a poor clinical outcome, including worse visual impairment in survivors. Study recommendations included evaluating each patient on a case-by-case basis but because of the poorer outcomes observed in abused patients, potentially considering earlier surgical intervention.

Another study [77] analyzed 213 abusive head trauma patients, including need for neurosurgical procedures and hospital costs. Fifty-eight patients underwent neurosurgical interventions that primarily included decompressive hemicraniectomies or burr hole procedures for subdural hemorrhage treatment. Additional procedures included placement of intracranial pressure monitors, external ventricular drains or internalized shunts for post-traumatic hydrocephalus, and subdural taps. This study also highlighted the overall increased healthcare costs for those patients requiring neurosurgical intervention.

Most patients suffering abusive head injury have poor outcomes, with nearly 20% of cases resulting in fatalities. Only about one-fifth may be left with mild to little appreciable impairment, leaving over half with moderate to severe neurologic, developmental, and/or visual impairments [78]. Predictive factors associated with more severe impairments include apnea or respiratory depression requiring intubation, cardiac arrest, acute ischemic-injury/infarction on imaging, as well as the presence of early post-traumatic seizures or status epilepticus [24,79,80,81]. The presence of hypoxic-ischemic injury with corresponding diffusion restriction on MRI has been found to be the most significant finding that correlates with poor clinical outcome and also contributes strongly to the presence of post-traumatic seizures [22,24,27,82]. Early post-traumatic seizures may be present at initial presentation but have their greatest severity within the second 24 h period [80]. Since they may go unrecognized in this population, prolonged or continuous electroencephalogram (EEG) may be warranted to aid with detection and subsequent treatment to avoid additional secondary insult to the brain [83,84].

Significant rates of visual impairment are also observed in abusive head-injured patients and correlate with retinal hemorrhage score as well as a more dismal overall outcome [85]. However, retinal hemorrhages may be absent in up to one-third of these patients [86]. Given that the complete extent of developmental, neuropsychiatric, and visual impairments may not be completely apparent at discharge, long-term, comprehensive follow-up, including post-injury clinics, is needed to aid with ongoing and future therapy and assistance [87]. Although many patients are left with moderate to severe impairments, abusive head trauma patients benefit from both inpatient and long-term rehabilitation, which may lead to significant functional achievements [79]. However, others may show progressive disability over time potentially due to existing comorbidities, including post-traumatic epilepsy, as well as additional social and emotional influences [85].

## 7. Conclusions

Abusive head injury is a serious form of child physical abuse and results in significant morbidity and mortality in this young pediatric population. As radiologists, we encounter a wide variety of cases each day, including cases of accidental trauma. Therefore, we are in a unique position to recognize when certain imaging findings stretch beyond what is typically expected. Therefore, radiologists need to be familiar not only with the more commonly known fracture patterns and presence of subdural hemorrhage that may be seen in these cases but also the patterns of parenchymal insults that may occur, especially those diffuse patterns of hemispheric involvement that may be subtle initially but may ultimately direct the radiologist to consider abusive head injury as a potential etiology.

## Data Availability

Not applicable.
